# Infrared Thermography Monitoring of Durum and Common Wheat for Adaptability Assessing and Yield Performance Prediction

**DOI:** 10.3390/plants13060836

**Published:** 2024-03-14

**Authors:** Massimo Rippa, Ida Di Mola, Lucia Ottaiano, Eugenio Cozzolino, Pasquale Mormile, Mauro Mori

**Affiliations:** 1 Institute of Applied Sciences and Intelligent Systems “E. Caianiello” of National Research Council of Italy (CNR ISASI), Via Campi Flegrei 34, 80072 Pozzuoli, Naples, Italy; p.mormile@isasi.cnr.it; 2 Department of Agricultural Sciences, University of Naples Federico II, 80055 Portici, Naples, Italy; ida.dimola@unina.it (I.D.M.); lucia.ottaiano@unina.it (L.O.); mori@unina.it (M.M.); 3 Council for Agricultural Research and Economics (CREA)—Research Center for Cereal and Industrial Crops, 81100 Caserta, Italy; eugenio.cozzolino@crea.gov.it

**Keywords:** infrared imaging, thermography, durum wheat, common wheat, index, yield, infrared monitoring

## Abstract

Wheat is one of the most cultivated cereals thanks to both its nutritional value and its versatility to technological transformation. Nevertheless, the growth and yield of wheat, as well as of the other food crops, can be strongly limited by many abiotic and biotic stress factors. To face this need, new methodological approaches are required to optimize wheat cultivation from both a qualitative and quantitative point of view. In this context, crop analysis based on imaging techniques has become an important tool in agriculture. Thermography is an appealing method that represents an outstanding approach in crop monitoring, as it is well suited to the emerging needs of the precision agriculture management strategies. In this work, we performed an on-field infrared monitoring of several durum and common wheat varieties to evaluate their adaptability to the internal Mediterranean area chosen for cultivation. Two new indices based on the thermal data useful to estimate the agronomical response of wheat subjected to natural stress conditions during different phenological stages of growth have been introduced. The comparison with some productive parameters collected at harvest highlighted the correlation of the indices with the wheat yield (ranging between *p* < 0.001 and *p* < 0.05), providing interesting information for their early prediction.

## 1. Introduction

Durum (*Triticum durum* Desf.) and common wheat (*Triticum aestivum* L.) are species with notable agricultural importance throughout much of the world, used as raw material for many foods, especially in the Mediterranean area [1,2,3]. Wheat represents one of the most cultivated cereals thanks to its nutritional value in the human diet and its versatility for the preparation of various foods [4,5]. However, to date, the yields are not enough to satisfy the increasing future demand; therefore, new agronomic and technological strategies are strongly required to optimize their production. In addition, yield can be strongly limited by several factors, such as lack of water, climatic conditions as well as the action of bacteria and fungi, all of which are the main abiotic and biotic stress factors to which wheat can be subjected [6,7]. One of the most limiting factors of crop development is temperature, which strictly affects its development and productivity. In fact, each phenological phase is characterized by an optimal range of temperature, over which the physiological processes are negatively affected [8,9,10,11,12]. On the other hand, the climate change scenarios forecast a global temperature increasing trend, with a higher frequency of heat waves and changes in rainfall patterns [13]. Indeed, even now, in Mediterranean environments, during the reproductive and grain-filling phase that occurs in the summer season, wheat grows under low rainfall and with heat waves [14]. Over time, farmers have planted different wheat genotypes to reduce the risk of failure and increase food security [15,16]; this practice gradually led to the development of various cereal landraces with defined genetic structures, mainly thanks to natural selection, which are adapted to the environmental growth conditions. This genetic diversification of crops can represent a strategy for the exploitation of biodiversity to cope with the various stress factors that can limit the cultivation of marginal lands, reducing their productivity [17]. Their ability to adapt to the specific pedological and climate conditions of the area in which they are grown can play a key role in determining the yield. In particular, for wheat, the use of old typical wheat varieties, which are more resilient, cultivated in purity or as mixtures, can also be a valid strategy to cope with climate change [8].

To counteract the problems caused by these stress factors, it is very important to develop high-throughput detection methods for stress level assessment in crops, which should be reliable, fast, simple, practical and economical. Digital crop analysis technologies have been widely used in recent decades as a means of optimizing crop management strategies. Imaging methods represent interesting established approaches to analyze both the state of health and the performance of crops under different environmental conditions [18,19,20,21]. Among these methods, infrared thermography (IT) is a well-known non-invasive and non-contact imaging technique that enables the analysis of large areas, which, in the last decade, has attracted great interest in agriculture and for food analysis [22,23,24,25]. In recent years, different IT approaches have been used to evaluate the physical and physiological characteristics of plants, among which are the heat capacity of the leaves, the local water content, transpiration rates, the water flow velocity and the response to UV irradiation [26,27,28,29,30,31,32,33,34]. Furthermore, it proved to be a valuable tool for the detection of different types of biotic and abiotic stresses in plants, adapting well to the emerging needs of precision agriculture management strategies [35,36,37,38,39,40,41,42]. 

The introduction of new indices for monitoring the plant status under limiting growth conditions (abiotic and/or biotic stress) is crucial in order to evaluate their effective adaptability and optimize the management strategies to increase both the quality of products and yield.

In general, crop surface temperature is driven by various factors, such as air and soil temperature, relative humidity, wind speed, light intensity, the presence of shadows as well as by plant features, such as their size. To take into account as much as possible the effects of these parameters (or some of them) which influence crop temperatures, numerous indices have been introduced and used for wheat crop monitoring. Elsayed et al. used the crop water stress index (CSWI) and normalized relative canopy temperature (NRCT) to analyze the water stress of wheat cultivars [43]. Banerjee et al. made use of the normalized sunlit shaded index (NSSI) for characterizing the moisture stress in wheat crops at different growth stages [44]. Wang et al. used the average canopy temperature (ACT), maximum temperature difference (MTD) and canopy temperature depression (CTD) to monitor the biotic stress induced on 25 wheat genotypes infected with *Zymoseptoria tritici* [45]. Romero-Bravo et al. used the stress degree day (SDD) to study the reliability for estimating grain yield and carbon isotope discrimination in various wheat genotypes [46]. Gracia-Romero et al. used the growing degree days (GDDs) to monitor the growth of 23 varieties of durum wheat [47].

Therefore, considering the importance of temperature for regular crop development, in this work, the proximal-scale infrared monitoring of ancient varieties of durum and common wheat, cultivated in purity and as mixtures in different percentages, was performed during the whole growth cycle and compared to modern varieties. The wheat varieties were cultivated in an internal hilly area of the Campania Region (Italy), characterized by climatic conditions typical of the Mediterranean basin. 

Our experimental approach allowed for the introduction of two indices useful to comparing the coverage of the monitored wheat varieties and for the identification of those ones subject to higher abiotic stress during the different phenological stages of growth. In particular, several varieties of durum and common wheat were cultivated in the Alto Tammaro area (Benevento, Campania Region—Italy). In summary, the aim of this experimental activity was as follows: (i) to select among modern and ancient varieties, and several mixtures of the latter, those varieties of durum and common wheat that show the best adaptability to the cultivation environment by the use of the new indices, and (ii) to compare these data with yield on-field measurements in order to verify their reliability in predictive terms.

## 2. Results and Discussion

### 2.1. Introduction to Experimental Analysis

Herein, we report on the experimental monitoring performed using the IT method to investigate the possibility of correlating thermal data with both the level of adaptability and the yield of different durum and common wheat varieties. The analysis was conducted using the passive thermography (PT) approach based on the acquisition of thermographic images of the crops recorded under natural conditions without the application of any external stimulus. Both species were monitored with three ancient varieties cultivated in purity, four mixtures at different percentage compositions and two modern varieties. The experimental design was arranged in randomized blocks with three replicates. Table 1 shows the information relating to the varieties.

**Figure 1:**
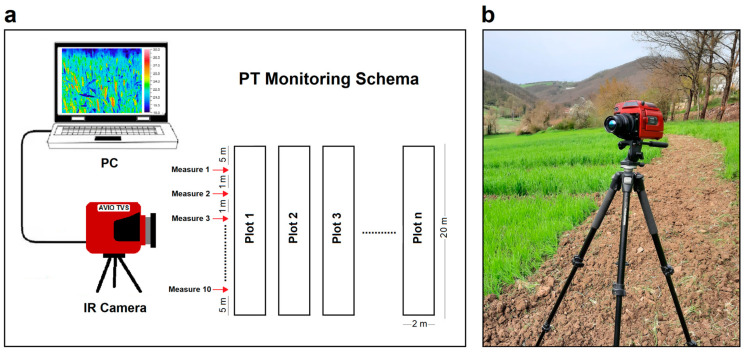
Passive thermography (PT) monitoring: (**a**) schema of the experimental design and (**b**) image of the infrared camera used in the field for thermal data recording.

The PT measurements were performed at the following four different phenological stages of the crops: stem elongation, the beginning of heading, milky–waxy ripening and full ripening (more details are provided in Section 3). In each phase, four measurement sessions were carried out for each species: two sessions per day for 2 consecutive days. In each measurement session, 30 thermal images of each crop (10 for each reply) were acquired in a proximal scale along the plot at about 1 m from each other. The temperatures of the first and last 5 m of the plots were most affected by the upper and lower edge and were not considered in the monitoring. A schema of the experimental design (Figure 1a) and an image of the infrared camera used in field for recording thermal data (Figure 1b) are shown in Figure 1.

### 2.2. Evaluation of the Wheat Stress Level Index

From the infrared images acquired, the average temperature of each wheat variety (*T_W_*) and the soil in their proximity (*T_S_*) was estimated at each monitored growth stage. We introduced a wheat stress index (*WSI*) using the following formal relationship:

*WSI* = (*T_S_* − *T_W_*)/*T_S_*

In all the measurements carried out, we found *T_S_* > *T_W_* > 0. Therefore, the index introduced is always positive, with values between 0 and 1 and, in general, the smaller the *T_W_*, the higher the value of the *WSI*. In the hypothesis that the *T_S_* − *T_W_* difference can be correlated to the crop stress level, lower *WSI* values can be associated with higher vegetation stress, while vice versa higher values with a lower stress level.

In comparison to the other indices present in the literature [22], e.g., the canopy temperature depression (CTD) [48,49,50,51], crop water stress index (CWSI) [50,51,52,53,54], canopy stress index (CSI) [55], Idso’s crop water stress index (ICWSI) [56], simplified stomatal conductance index (SSCI) [57], temperature ratio index (TRI) [58], maximum temperature difference (MTD) [59] or normalized relative canopy temperature (NRCT) [60], normalized with respect to the air temperature (CTD, TRI), dry/wet references (CWSI, SSCI), vapor pressure deficit (CSI) or temperature variations on the canopy (ICWSI, MTD), the new index, the *WSI*, is normalized with respect to the temperature of the soil, *T_S_*, in proximity to each specific plot. This choice was based on the following two main considerations: (1) *T_S_* better describes the microclimatic conditions at the crop level, also taking into account the eventual differences in both irradiation (the presence of shaded areas) or soil moisture; (2) unlike measuring the air temperature or other references (as *T_dry_*, *T_wet_* or *T_vpd_*), which requires additional instruments and measurements, *T_S_* can be extrapolated directly from the acquired thermal frames, thus facilitating the design of automatic and remote analysis systems. 

**Figure 2:**
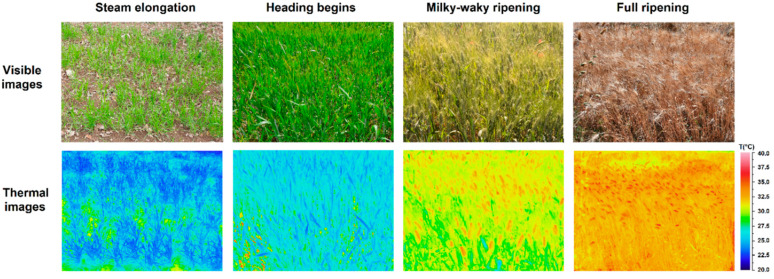
Analyses of the crop stress level: visible and thermal images acquired in four growth states for one of the varieties investigated.

As an example of the measurements carried out in field, in Figure 2, for one of the varieties investigated, both the visible and thermal images acquired in the four phenological phases considered for monitoring are shown.

Table 2 reports the WSI values measured in the four phenological stages per each durum and common wheat variety, as well as the mean values per each variety (*WSI_m_*) and each phenological stage (*PP-WSI_m_*). The *WSI_m_* can be considered as an indicator of the average level of stress exhibited by a crop throughout the growth period. For durum wheat, the *WSI_m_* ranged between 0.15 (MixD4) and 0.29 as recorded for Svevo that, according to our assumptions, represents the variety that was characterized by the lowest level of stress and, therefore, the greatest adaptability to cultivation conditions. In the case of common wheat, the *WSI_m_* ranged between 0.15 (MixC1 and MixC3) and 0.26, as recorded for Alteo. 

**Figure 3:**
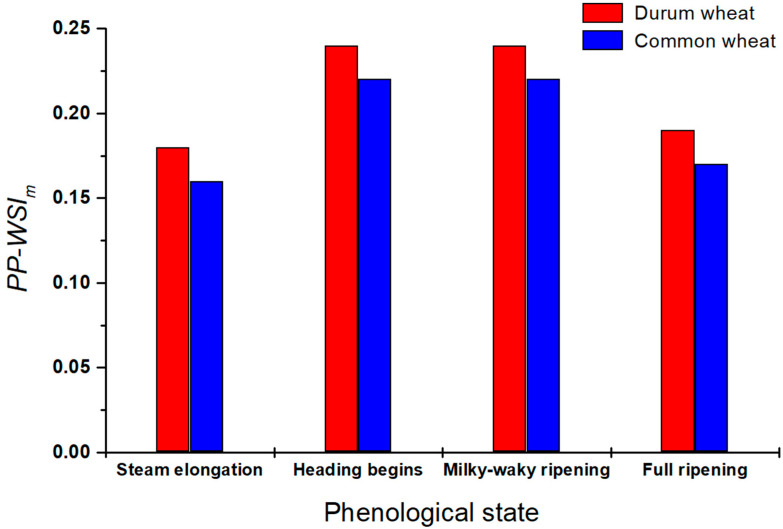
Graph of the *PP-WSI_m_* estimated for the four phenological states monitored for both durum wheat (red columns) and common wheat (blue columns).

The *PP-WSI_m_* values of the four monitored phenological stages for both durum and common wheat are also reported in Figure 3. This parameter highlights a bell trend, with the lower values recorded at the stem elongation and full ripening (initial and final stages of the crop cycle) and the higher values in the central phases (heading begins and milky–waxy ripening). Therefore, it seems that the phase of fast vegetative growth (stem elongation) and that of the full ripening of grains are the most sensitive to stress conditions. Thus, these crop phases of both durum and common wheat require more monitoring by farmers and an appropriate agricultural management in order to avoid yield losses. 

As regards the species’ response, interestingly, in all stages, durum wheat showed higher values of the *PP-WSI_m_* than the common wheat and, hence, a lower average level of stress. These results are probably due to the genetic differences between the two species, but also to their different adaptability to cultivation in marginal soils (for example, hilly), where durum wheat has always historically been cultivated. In addition, the microclimatic conditions of the two sites had also probably affected the response of the two species, since the cultivation site of the durum wheat was more ventilated and less humid than that of the common wheat. 

### 2.3. Evaluation of the Wheat Cover Index

The infrared images of the crops acquired in the stem elongation phenological stage were used to evaluate a coverage index of the tested wheat varieties. To estimate this parameter, a simple post-processing analysis algorithm was applied to the recorded data. The approach used is based on the estimation of the number of pixels relating to the wheat (*P_W_*) and to the soil (*P_S_*) exhibited in the acquired thermal images. The separation of the two classes of pixels was carried out considering the temperature distribution diagrams associated with the images. The analysis of the diagrams can allow us to identify a temperature threshold value, *T_TH_*, useful for discriminating the temperature distribution of the wheat and that of the soil. In all our measurements, pixels, *P_W_*, relating to the wheat were characterized by a temperature *T_w_* < *T_TH_*, while those relating to soil, *P_S_*, by a temperature *T_S_* > *T_TH_*. Therefore, from these estimations, a wheat cover index (*WCI*) has been calculated using the following formal relationship:

*WCI* = *P_W_*/(*P_W_* + *P_S_*)

**Figure 4:**
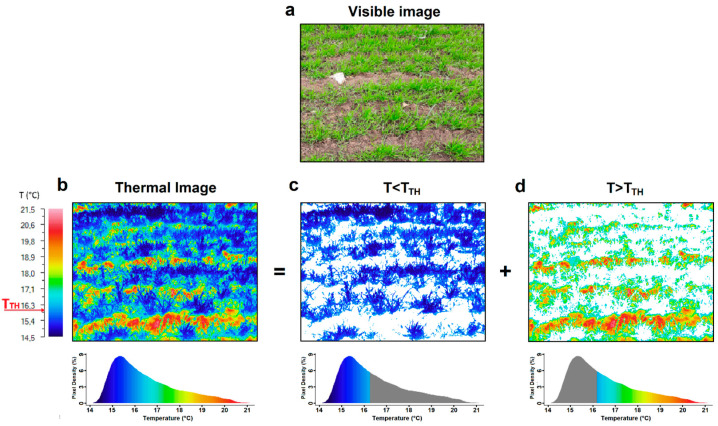
Wheat cover index estimation: (**a**) visible image of a wheat cultivation clod, (**b**) the corresponding thermographic image, (**c**) the image of the pixels relating to the wheat and (**d**) the image of the pixels relating to the soil. Below each thermal image, the corresponding temperature distribution diagram is shown. The case reported in figure was estimated as T_TH_ = 16.2 °C.

Notably, this index is always positive; it can assume values between 0 and 1, and the higher the value, the higher the wheat coverage. In Figure 4, as an example, are shown a visible image of a wheat cultivation area (Figure 4a), the corresponding thermal image (Figure 4b), two of its representations, in which only the pixels associated with the vegetation (Figure 4c) or with the soil (Figure 4d) are reported, and their respective temperature distribution diagrams used for the *T_TH_* estimation.

The choice of an adequate *T_TH_* value is a critical point that must be performed carefully to avoid overestimates or underestimates of the *WCI* introduced. In our cases, the temperature diagrams were characterized either by the presence of a double peak associated, respectively, with the vegetation and the soil, or by a continuous trend, as in the case of Figure 4b. In the case of a double peak, a reasonable choice may be to consider *T_TH_* as the average value of the peak temperatures, while in the case of a continuous trend, the comparison with the visible image can help to select its most appropriate value that maximizes the separation of the two temperature classes. It must be said that, in all cases, this type of evaluation based on the choice of a *T_TH_* is subject to an intrinsic error due to the presence of pixels in the diagram relating to the vegetation having *T_W_* > *T_TH_* and pixels relating to the soil having *T_S_* < *T_TH_*. However, in the reasonable hypothesis that the number of such pixels is approximately equal and negligible with respect to both *P_W_* and *P_S_*, the proposed index allows for an estimation of the percentage of the soil coverage of the wheat crops and a comparison of the different types of plots considered. Furthermore, it is important to point out that this type of analysis and comparison can only be carried out in the initial growth phase of the crop, when the small size of the vegetal part does not completely cover the view of the soil. For each crop, the described analysis approach was applied to all thermal images acquired in the phenological state taken into account, and a final *WCI* value was estimated by averaging. In Table 3, the average *WCI* values estimated for all wheat varieties monitored (durum and common) are reported.

The *WCI_m_* calculated are in the range 61–93% for both wheat species, with the highest values (93%) found in the case of the Senatore Cappelli (SC) variety for durum wheat and the Gentilrosso (GR) variety for common wheat.

### 2.4. Yield and Its Component Parameters

For durum wheat, the yield, the number of culms and spikes per square meter and the total biomass were significantly affected by the variety factor (Table 4), while, for common wheat, all parameters except the total biomass were statistically affected by the variety (Table 5). As for durum wheat, the modern varieties SV and PG showed the highest yields, with 3.5 and 2.4 t ha^−1^, respectively. Among the ancient varieties and their mixtures, only SC, SL and MixD2 reached 2.0 t ha^−1^, but only SC was not different from SV and PG. In terms of the culms and spikes per square meter, the differences between the varieties were not marked, and only MixD3 showed the lowest values for both parameters, but it was significantly different only from MixD2, and for MixD1, MixD2 and MixD4 for the number of culms and spikes m^−2^, respectively (Table 4). Finally, the lowest value of the total biomass was recorded for the modern Variety Pigreco, but it was different only from Senatore Cappelli (Table 4). 

As regards the common wheat, the modern variety Alteo had the best yield performance with 3.0 t ha^−1^, but it was not different from Axum, Risciola, Gentilrosso and MixC4, while the other three mixtures produced, by mean, about half of Alteo. The highest yield of Alteo was due to the significantly higher number of culms and spikes per square meter, also if it was significantly different only from MixC1, MixC2 and MixC3, respectively (Table 5). 

### 2.5. Data Correlation

**Figure 5:**
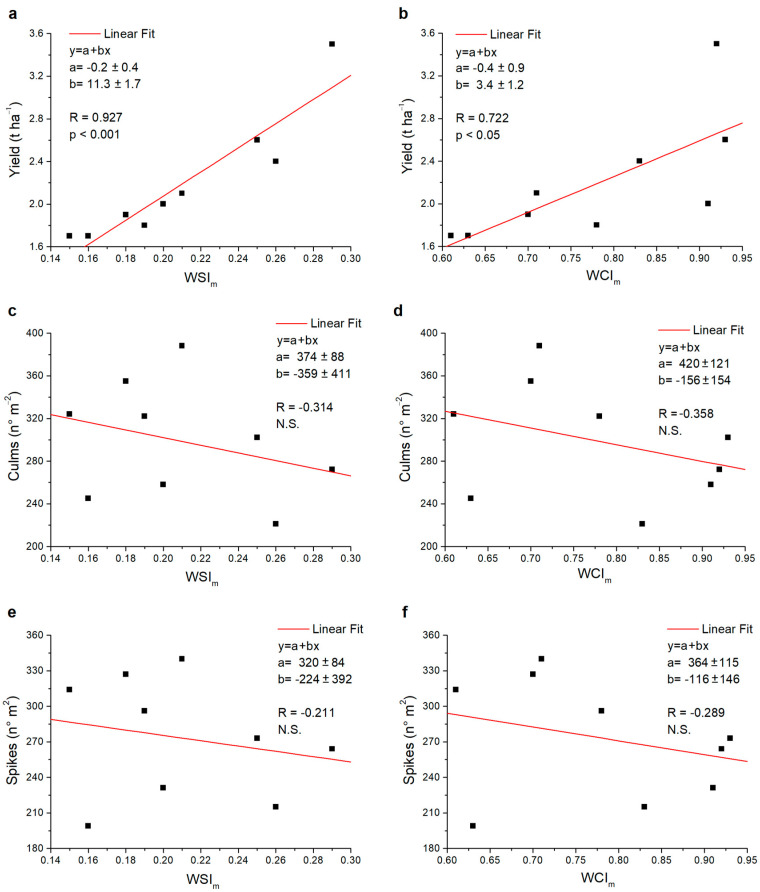
Scatter plots of the values relating to the durum wheat varieties investigated: (**a**) *WSI_m_* versus yield, (**b**) *WCI_m_* versus yield, (**c**) *WSI_m_* versus culms, (**d**) *WCI_m_* versus culms, (**e**) *WSI_m_* versus spikes and (**f**) *WCI_m_* versus spikes. In each graph, the fit values *a* and *b*, the Pearson correlation index *R* and, respectively, the significance (*p*) or non-significance (N.S.) found are reported.

To evaluate the predictive potential of the two new indices, *WSI* and *WCI*, we tested the correlations between the values obtained for the two indices and the measured yield parameters. Figure 5 shows the correlations between the *WSI_m_* (Figure 5a,c,e) and the *WCI_m_* (Figure 5b,d,f) and the yield, as well as the number of culms and the number of spikes of the tested durum wheat varieties. For each graph, a linear fit, *y* = *a* + *bx*, was performed, and the Pearson correlation index, *R*, was calculated. Subsequently, the *p*-significance of the data was verified according to the t-test under non-directional hypotheses. The numerical results obtained are reported in the respective graphs. The results of the analysis show a significant correlation of both indices only with the yield parameter, with the significance level of the *WSI_m_* (*p* < 0.001) higher than that of the *WCI_m_* (*p* < 0.05) (Figure 5a,b). Conversely, no significant correlation was found for the two indices with the number of culms and spikes (Figure 5c–f).

**Figure 6:**
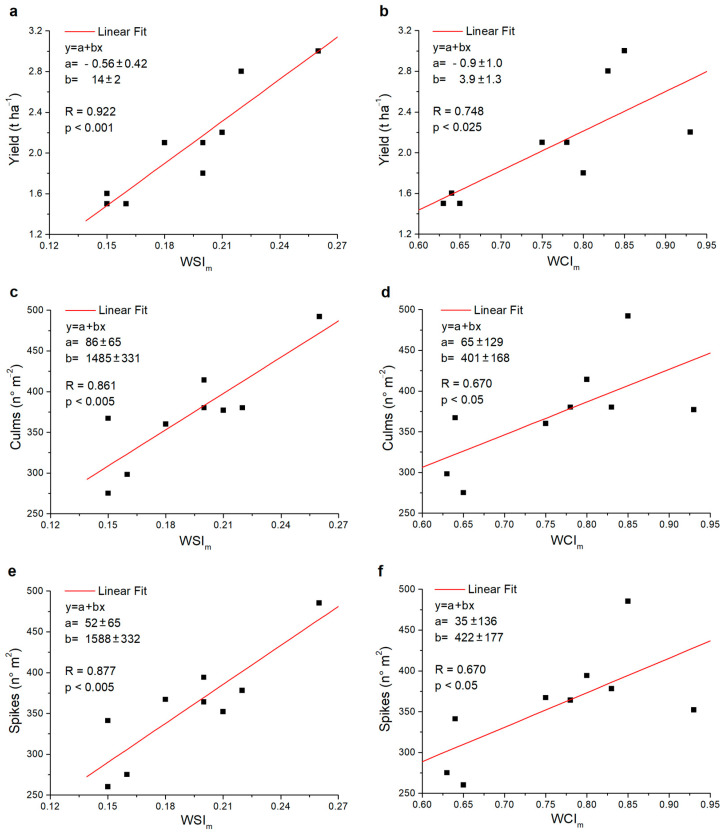
Scatter plots of the values relating to the common wheat varieties investigated: (**a**) *WSI_m_* versus yield, (**b**) *WCI_m_* versus yield, (**c**) *WSI_m_* versus culms, (**d**) *WCI_m_* versus culms, (**e**) *WSI_m_* versus spikes and (**f**) *WCI_m_* versus spikes. In each graph, the fit values *a* and *b*, the Pearson correlation index *R* and, respectively, the significance (*p*) or non-significance (N.S.) found are reported.

The same analysis was also performed for the common wheat data, and the graphs are reported in Figure 6. Interestingly, in this case, all the correlations between the two indices and yield, as well as the number of culms and spikes, were significant, and they ranged between *p* < 0.001 and *p* < 0.05. In addition, the significance level of the *WSI_m_* was always higher than that of the *WCI_m_*, ranging between *p* < 0.001 and *p* < 0.005 and between *p* < 0.025 and *p* < 0.05 for the *WSI_m_* and *WCI_m_*, respectively.

**Figure 7:**
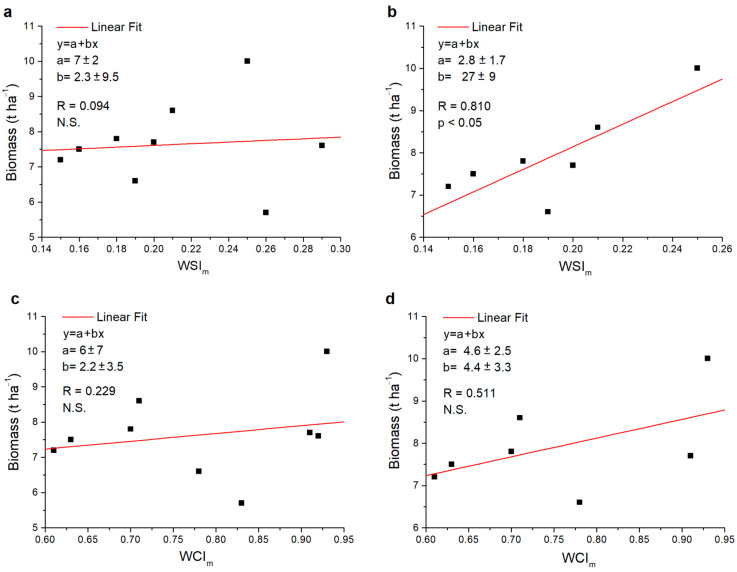
Scatter plots of the values relating to the durum wheat varieties investigated: (**a**) *WSI_m_* versus biomass, (**b**) *WSI_m_* versus biomass not considering the modern varieties PG and SV, (**c**) *WCI_m_* versus biomass and (**d**) *WCI_m_* versus biomass not considering the modern varieties PG and SV. In each graph, the fit values *a* and *b*, the Pearson correlation index *R* and, respectively, the significance (*p*) or non-significance (N.S.) found are reported.

When the *WSI_m_* was correlated to the total biomass, for durum wheat, the significance of this correlation was strongly affected by the presence or absence of the two modern varieties among the data; indeed, it was not significant when the index was correlated with all the varieties (Figure 7a), and significant when excluding the two modern varieties, PG and SV (Figure 7b). As regards the *WCI_m_*, instead, the correlation was never significant, irrespective of the considered varieties (Figure 7c,d). Moreover, when all varieties were considered, the R values for both indices were very low (Figure 7a,c); by excluding the PG and SV varieties from the analysis, the R values obtained for both the *WSI_m_* and *WCI_m_* increased considerably (Figure 7b,d). 

**Figure 8:**
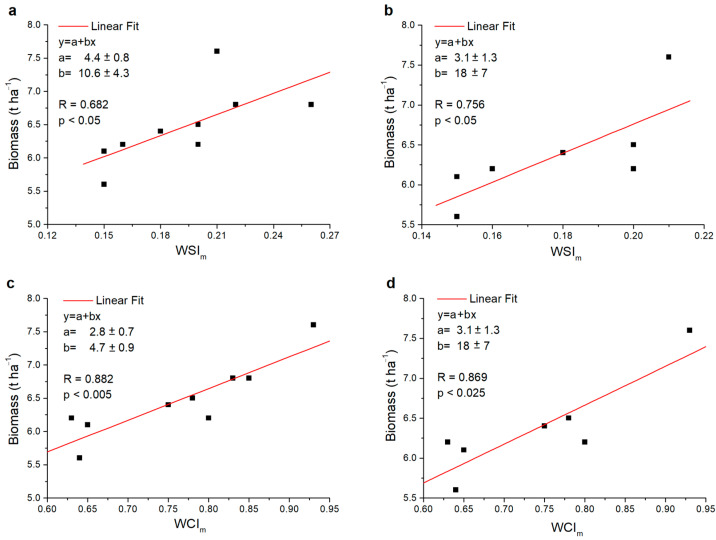
Scatter plots of the values relating to the common wheat varieties investigated: (**a**) *WSI_m_* versus biomass, (**b**) *WSI_m_* versus biomass not considering the modern varieties PG and SV, (**c**) *WCI_m_* versus biomass and (**d**) *WCI_m_* versus biomass not considering the modern varieties AX and AL. In each graph the fit values *a* and *b*, the Pearson correlation index *R* and, respectively, the significance (*p*) or non-significance (N.S.) found are reported.

A different result was found in the common wheat; in fact, in this case, the correlation was always significant regardless of whether the data of the modern varieties were considered or not, and with a significance ranging from *p* < 0.05 to *p* < 0.005 (Figure 8). In particular, by excluding the two modern varieties, an increase in *R* was observed in the case of the *WSI_m_*, but not for the *WCI_m_.* These variations may be attributable to normal statistical fluctuations in the results that occur when data are added or subtracted to the analysis. However, in the case of the *WCI_m_*, the latter led to light variations in the significance level found.

Probably, the different behavior of durum and common wheat is due to the different genetic improvement to which the two species have been subjected over the years. In fact, the traditional varieties of durum wheat used in our research (Senatore Cappelli, Marzellina and Saragolla lucana), but not only these, are generally high-size varieties with a greater biomass (8.1 t ha^−1^, the mean value of old varieties, vs. 6.6, the mean value of modern varieties; Table 4) and plant height. Since these morphological traits increase the risk of wheat lodging, with a consequent loss of yield, in the last decades, geneticists have notably reduced the plant size and improved the yield traits. Instead, for common wheat, the variation between varieties/mixtures in terms of biomass were very low: 6.8, 6.5 and 6.8 t ha^−1^ mean values of the ancient, mixture and modern varieties, respectively (Table 5). Therefore, a greater genetic similarity among the old and modern varieties can be assumed, at least for those ones tested in the current research, and this can explain why the correlation was also significant when the modern varieties were included in the analysis. 

## 3. Materials and Methods

### 3.1. Experimental Design, Plant Materials and Crop Management

The experiment was carried out during the growing seasons 2021–2022 on two private organic farms located in the “Alto Tammaro” area (Benevento, Campania Region—Italy): the “Paolucci” farm, located in Colle Sannita (latitude: 41°22′48.5″ N, longitude: 14° 52′09.5″ E, altitude: 695 m), and the “Di Iourio” farm, located in Castelpagano (latitude: 41°25′50.7″ N, longitude: 14°48′15.1″ E, altitude: 805 m). In both farms, the experimental design was a comparison of 3 ancient wheat varieties, 4 with a mixture at different percentages and 2 modern varieties. 

In particular, at the Paolucci farm, the durum wheat was tested. The ancient varieties were as follows: (i) Senatore Cappelli—SC; (ii) Marzellina MZ; (iii) Saragolla Lucana—SL; the four mixtures were made with the following percentages: (i) 33% SC + 33% MZ + 33% SL; (ii) 50% SC + 25% MZ + 25% SL; (iii) 25% SC + 50% MZ + 25% SL; 25% SC + 25% MZ + 50% SL, hereinafter referred to as MixD1, MixD2, MixD3 and MixD4; the 2 modern varieties were as follows: (i) Svevo—SV; (ii) Pigreco—PG.

At the Di Iourio farm, the common wheat was tested. The ancient varieties were as follows: (i) Risciola—RS; (ii) Romanella—RM; (iii) Gentilrosso—GR; the four mixtures were made with the following percentages: (i) 33% RS + 33% RM + 33% GR; (ii) 50% RS + 25% RM + 25% GR; (iii) 25% RS + 50% RM + 25% GR; (iv) 25% RS + 25% RM + 50% GR, hereinafter referred to as MixC1, MixC2, MixC3 and MixC4; the 2 modern varieties were as follows: (i) Axum—AX; (ii) Alteo—AL.

All wheat varieties and their mixtures investigated are reported in Table 1.

In both farms, the design was arranged in randomized blocks with 3 replicates for a total of 27 plots in each farm, where each plot was 40 m^2^. The sowing was performed on 22 December 2021, with a density of 450 seeds per square meter. According to organic cultivation, no fertilization nor interventions for weed and pathogen control were made. The harvests were performed on 11 July 2022 in both farms.

### 3.2. Yield and Yield Parameter Measurements

In both farms, at the harvest, the following measurements were made: grain yield and total biomass (both expressed as tons per hectare), and number of culms and spikes per square meter. 

### 3.3. Infrared Thermography Measurements

Infrared measurements were performed using an LWIR AVIO TVS500 (Nippon Avionics Co., Yokohama, Japan) with an uncooled microbolometric detector (spectral range 8–14 μm, FPA 320 × 240 pixels and NETD ~60 mK at 25 °C) mounting a 22 mm focal lens with an IFOV 1.68 mrad. The commercial software IRT Analyzer ver. 4.8 (GRAYESS Inc., Bradenton, FL, USA), with which the camera was supplied, was used for monitoring the temperature in real-time and for basic operations. Measurements were performed at the following four different phenological stages of the crops: stem elongation (21–22 April 2022), beginning of heading (26–27 May 2022), milky–waxy ripening (13–14 June 2022) and full ripening (29–30 June 2022). In each phase, for two consecutive days, two measurement sessions were carried out in the same time interval (10–12 a.m. and 15–17 p.m.). In each session, 20 thermal images of each crop were recorded along the plot approximately 1 m from each other, excluding the first and last 5 m of the plot. A schema of the experimental design is shown in Figure 1a.

### 3.4. Statistical Analysis

All productive data were subjected to statistical analysis by one-way ANOVA with SPSS (version 22, Chicago, IL, USA) and means were separated using Tukey’s test at *p* ≤ 0.05. The *t*-test to verify the correlations between the yield measurements and the two indices were conducted on the data set.

## 4. Conclusions

In this work, the IT technique was employed to monitor and compare the thermal state of different varieties of durum and common wheat, aiming to assess their adaptability and to make a prediction of the yield performance. Two new indices, the *WSI* and *WCI*, were introduced and compared with the yield parameters, highlighting interesting correlations. The *WSI* can be estimated during the whole cultivation period and can provide indications on the stress level of the crops at each growth phase. The *WCI* can be estimated in an initial growth phase (stem elongation) and does not take into account the successive cycle phases. 

Our preliminary findings highlight that stem elongation and full ripening represent the phenological phases susceptible to higher stress levels, which therefore require greater control and monitoring by the farmer and adequate optimization of the management strategy. Interestingly, in all stages, durum wheat always showed a lower average stress level than common wheat. The *WSI* provides useful feedback to the farmer for a more adequate agricultural management and, moreover, allows a good yield prediction. The *WCI* is less reliable in predicting the final yield than the *WSI*, but it represents an interesting index to have for a first indication on the harvest at an early stage.

Finally, these two indices can provide useful information on the adaptability and yield prediction of durum and common wheat and, in addition, they can be employed to develop new proximal and remote sensing systems. The results achieved are very interesting, but further research is needed in order to confirm them also in other pedo-climatic conditions. Furthermore, future experiments will be carried out to test the versatility of the indices for monitoring other crops as well.

## Figures and Tables

**Table 1:** List of durum and common wheat varieties and their mixtures monitored with the passive thermography method.

Durum Wheat		Common Wheat	
Variety	Composition	Variety	Composition
Senatore Cappelli (SC)	pure	Risciola (RS)	pure
Marzellina (MZ)	pure	Romanella (RM)	pure
Saragolla Lucana (SL)	pure	Gentilrosso (GR)	pure
MixD1	33% SC–33% MZ–33% SL	MixC1	33% RS–33% RM–33% GR
MixD2	50% SC–25% MZ–25% SL	MixC2	50% RS–25% RM–25% GR
MixD3	25% SC–50% MZ–25% SL	MixC3	25% RS–50% RM–25% GR
MixD4	25% SC–25% MZ–50% SL	MixC4	25% RS–25% RM–50% GR
Pigreco (PG)	pure	Axum (AX)	pure
Svevo (SV)	pure	Alteo (AL)	pure

**Table 2:** *WSI* values measured for the durum and common wheat varieties investigated in the following four phenological stages monitored: stem elongation (S.E.), heading begins (H.B.), milky–waxy ripening (M.W.R.) and full ripening (F.R.). *WSI_m_* and *PP-WSI_m_* are the average values of the WSI calculated considering the value in the rows (for each variety) or in the columns (for each phenological phase), respectively. For all values, a maximum error σ = ±0.01 was estimated.

Durum Wheat						Common Wheat					
Variety	*WSI*				*WSI_m_*	Variety	*WSI*				*WSI_m_*
	S.E.	H.B.	M.W.R.	F.R.			S.E.	H.B.	M.W.R.	F.R.	
SC	0.21	0.31	0.27	0.21	0.25	RS	0.18	0.23	0.23	0.17	0.20
MZ	0.16	0.19	0.23	0.17	0.19	RM	0.17	0.25	0.21	0.18	0.20
SL	0.18	0.22	0.23	0.19	0.20	GR	0.19	0.22	0.25	0.17	0.21
MixD1	0.14	0.20	0.21	0.16	0.18	MixC1	0.12	0.18	0.17	0.13	0.15
MixD2	0.18	0.24	0.24	0.19	0.21	MixC2	0.13	0.19	0.18	0.14	0.16
MixD3	0.15	0.19	0.17	0.14	0.16	MixC3	0.12	0.17	0.16	0.15	0.15
MixD4	0.14	0.17	0.18	0.13	0.15	MixC4	0.16	0.21	0.19	0.17	0.18
PG	0.23	0.31	0.28	0.23	0.26	AX	0.18	0.21	0.30	0.19	0.22
SV	0.24	0.32	0.33	0.26	0.29	AL	0.23	0.32	0.27	0.21	0.26
*PP-WSI_m_*	0.18	0.24	0.24	0.19		*PP-WSI_m_*	0.16	0.22	0.22	0.17	

**Table 3:** Average WCI values estimated for all wheat varieties monitored from the thermal data acquired in the stem elongation phenological stage.

Durum Wheat		Common Wheat	
Variety	*WCI_m_*	Variety	*WCI_m_*
SC	0.93 ± 0.05	RS	0.78 ± 0.04
MZ	0.78 ± 0.04	RM	0.80 ± 0.04
SL	0.91 ± 0.04	GR	0.93 ± 0.05
MixD1	0.70 ± 0.03	MixC1	0.65 ± 0.03
MixD2	0.71 ± 0.03	MixC2	0.63 ± 0.04
MixD3	0.63 ± 0.04	MixC3	0.64 ± 0.04
MixD4	0.61 ± 0.04	MixC4	0.75 ± 0.04
PG	0.83 ± 0.04	AX	0.83 ± 0.05
SV	0.92 ± 0.05	AL	0.85 ± 0.04

**Table 4:** Yield and yield components (number of culms and spikes per square meter) and total biomass of the nine durum wheat varieties/mixtures.

Variety	Yield (t ha^−1^)	Culms (n° m^2^)	Spikes (n° m^2^)	Total Biomass (t ha^−1^)
SC	2.6 ± 0.2 ab	302 ± 15 ab	273 ± 8 ab	10.0 ± 1.0 a
MZ	1.8 ± 0.2 b	322 ± 32 ab	296 ± 24 ab	6.6 ± 0.6 ab
SL	2.0 ± 0.2 b	258 ± 25 ab	231 ± 11 ab	7.7 ± 0.6 ab
MixD1	1.9 ± 0.3 b	355 ± 16 ab	327 ± 11 a	7.8 ± 0.9 ab
MixD2	2.1 ± 0.4 b	388 ± 39 a	340 ± 40 a	8.6 ± 0.8 ab
MixD3	1.7 ± 0.3 b	245 ± 30 b	199 ± 54 b	7.5 ± 0.5 ab
MixD4	1.7 ± 0.3 b	324 ± 40 ab	314 ± 33 a	7.2 ± 1.4 ab
PG	2.4 ± 0.2 ab	221 ± 29 b	215 ± 31 ab	5.7 ± 0.6 b
SV	3.5 ± 0.3 a	272 ± 10 ab	264 ± 3 ab	7.6 ± 0.9 ab
Significance	**	*	*	*

* and ** refer to significant at *p* < 0.05 and *p* < 0.01. In each column, different letters indicate significant differences.

**Table 5:** Yield and yield components (number of culms and spikes per square meter) and total biomass of the nine common wheat varieties/mixtures.

Variety	Yield (t ha^−1^)	Culms (n° m^2^)	Spikes (n° m^2^)	Total Biomass (t ha^−1^)
RS	2.1 ± 0.2 ac	380 ± 43 ab	364 ± 36 ab	6.5 ± 0.8 ab
RM	1.8 ± 0.1 bc	414 ± 34 ab	394 ± 35 ab	6.2 ± 0.1 ab
GR	2.2 ± 0.2 ac	377 ± 46 ab	352 ± 51 ab	7.7 ± 0.5 ab
MixC1	1.5 ± 0.1 c	275 ± 22 b	260 ± 20 b	6.1 ± 0.2 ab
MixC2	1.5 ± 0.2 c	298 ± 41 ab	275 ± 46 b	6.2 ± 0.5 ab
MixC3	1.6 ± 0.3 c	367 ± 41 ab	341 ± 46 ab	5.6 ± 0.5 b
MixC4	2.1 ± 0.1 ac	360 ± 34 ab	367 ± 46 ab	6.4 ± 0.1 ab
AX	2.8 ± 0.2 ab	380 ± 55 ab	378 ± 46 ab	6.8 ± 0.6 ab
AL	3.0 ± 0.3 a	492 ± 23 a	485 ± 46 a	6.8 ± 0.5 ab
Significance	**	*	*	*

* and ** refer to significant at *p* < 0.05 and *p* < 0.01. In each column, different letters indicate significant differences.

## Data Availability

The raw data supporting the conclusions of this article will be made available by the authors on request. The data are not publicly available mainly due to its size and quantity (numerous infrared image sequences).

## References

[1] Mangini G., Margiotta B., Marcotuli I., Signorile M.A., Gadaleta A., Blanco A. (2017). Genetic diversity and phenetic analysis in wheat (*Triticum turgidum* subsp. *durum* and *Triticum aestivum* subsp. *aestivum*) landraces based on SNP markers. Genet. Resour. Crop Evol..

[2] Alfonzo A., Sicard D., Di Miceli G., Guezenec S., Settanni L. (2020). Ecology of yeasts associated with kernels of several durum wheat genotypes and their role in co-culture with Saccharomyces cerevisiae during dough leavening. Food Microbiol..

[3] Peña-Bautista R.J., Hernandez-Espinosa N., Jones J.M., Guzmán C., Braun H.J. (2017). CIMMYT series on carbohydrates, wheat, grains, and health: Wheat-based foods: Their global and regional importance in the food supply, nutrition, and health. Cereal Foods World.

[4] Pomeranz Y., Pomeranz Y. (1987). Bread around the world. Modern Cereal Science and Technology.

[5] Braun H.J., Atlin G., Payne T., Reynolds C.R.P. (2010). Multi-location testing as a tool to identify plant response to global climate change. Climate Change and Crop Production.

[6] Afzal F., Khalil S., Gul A., Farooq A., Ali H., Nisar S., Sarfraz B., Shehzadi K.J., Mujueeb-Kazi A., Hakeem K. (2015). Bread Wheat (*Triticum aestivum* L.) Under Biotic and Abiotic Stresses: An Overview. Crop Production and Global Environmental Issues.

[7] Akter N., Rafiqul Islam M. (2017). Heat stress effects and management in wheat. A review. Agron. Sustain. Dev..

[8] Ottaiano L., Di Mola I., Cozzolino E., Mori M. (2022). Preliminary Results of the Use of Sowing Time and Variety Choice as Techniques of Adaptability of Durum Wheat (*Triticum durum* Desf.) to Temperature Increases. Sustainability.

[9] Ayed S., Othmani A., Chaieb N., Rezgui M., Ben Younes M. (2016). Relation between agro-meteorological indices, heading date and biological/grain yield of durum wheat genotypes. J. Res. Agric. Anim. Sci..

[10] Tack J., Barkley A., Nalley L.L. (2015). Effect of warming temperatures on US wheat yields. Proc. Natl. Acad. Sci. USA.

[11] Salazar-Gutierrez M.R., Johnson J., Chaves-Cordoba B., Hoogenboom G. (2013). Relationship of base temperature to development of winter wheat. Int. J. Plant Prod..

[12] Asseng S., Foster I., Turner N.C. (2011). The impact of temperature variability on wheat yields. Glob. Chang. Biol..

[13] Hartmann D.L., Klein Tank A.M.G., Rusticucci M., Alexander L.V., Brön-nimann S., Charabi Y., Dentener F.J., Dlugokencky E.J., Easterling D.R., Kaplan A. (2013). Observations: Atmosphere and surface. Climate Change 2013: The Physical Science Basis. Contribution of Working Group I to the Fifth Assessment Report of the Intergovernmental Panel on Climate Change.

[14] Lyon C., Saupe E.E., Smith C.J., Hill D.J., Beckerman A.P., Stringer L.C., Marchant R., McKay J., Burke A., O’Higgins P. (2022). Climate change research and action must look beyond 2100. Glob. Chang. Biol..

[15] Jaradat A.A. (2006). Phenotypic divergence in the meta-population of the Hourani durum wheat landrace. J. Food Agric. Environ..

[16] Jaradat A.A. (2017). Wheat Landraces: A Mini Review. Emir. J. Food Agric..

[17] Carvajal-Yepes M., Cardwell K., Nelson A., Garrett K.A., Giovani B., Saunders D.G.O., Kamoun S., Legg J.P., Verdier V., Lessel J. (2019). A global surveillance system for crop diseases. Science.

[18] Li L., Zhang Q., Huang D. (2014). A review of imaging techniques for plant phenotyping. Sensors.

[19] Karmakar P., Teng S.W., Murshed M., Pang S., Li Y., Lin H. (2024). Crop monitoring by multimodal remote sensing: A review. Remote Sens. Appl. Soc. Environ..

[20] Polder G., Dieleman J.A., Hageraats S., Meinen E. (2024). Imaging spectroscopy for monitoring the crop status of tomato plants. Comput. Electron. Agric..

[21] Li D., Li C., Yao Y., Li M., Liu L. (2020). Modern imaging techniques in plant nutrition analysis: A review. Comput. Electron. Agric..

[22] Pineda M., Barón M., Pérez-Bueno M.L. (2021). Thermal imaging for plant stress detection and phenotyping. Remote Sens..

[23] Ishimwe R., Abutaleb K., Ahmed F. (2014). Applications of thermal imaging in agriculture—A review. Adv. Remote Sens..

[24] Vadivambal R., Jayas D.S. (2011). Applications of thermal imaging in agriculture and food industry—A review. Food Bioprocess Technol..

[25] Aryalekshmi B.N., Biradar R.C., Mohammed Ahamed J. (2019). Thermal Imaging Techniques in Agricultural Applications. Int. J. Innov. Technol. Explor. Eng..

[26] Capraro A.C.W., Steppe K., Van Asten P.J.A., Laderach P., Jassogne L.T.P., Grab S.W. (2017). Application of thermography for monitoring stomatal conductance of Coffea Arabica under different shading systems. Sci. Total Environ..

[27] Guilioni L., Jones H.G., Leinonen I., Lhomme J.P. (2008). On the relationships between stomatal resistance and leaf temperatures in thermography. Agric. For. Meteorol..

[28] Garbea C.S., Schurrb U., Jähnea B. (2002). Thermographic measurements on plant leaves. Thermosense XXIV.

[29] Rippa M., Battaglia V., Cermola M., Sicignano M., Lahoz E., Mormile P. (2022). Monitoring of the copper persistence on plant leaves using pulsed thermography. Environ. Monit. Assess..

[30] Oerke E.C., Fröhling P., Steiner U. (2011). Thermographic assessment of scab disease on apple leaves. Precis. Agric..

[31] Cohen Y., Alchanatis V., Sela E., Saranga Y., Cohen S., Meron S., Bosak A., Tsipris J., Ostrovsky V., Orolov V. (2015). Crop water status estimation using thermography: Multi-year model development using ground-based thermal images. Precis. Agric..

[32] Rippa M., Ambrosone A., Leone A., Mormile P. (2020). Active thermography for real time monitoring of UV-B plant interactions. J. Photochem. Photobiol. B Biol..

[33] Blonquist J.M., Norman J.M., Bugbee B. (2009). Automated measurement of canopy stomatal conductance based on infrared temperature. Agric. For. Meteorol..

[34] Bajons P., Klinger G., Schlosser V. (2005). Determination of stomatal conductance by means of infrared thermography. Infrared Phys. Technol..

[35] Zhou Z., Diverres G., Kang C., Thapa S., Karkee M., Zhang Q., Keller M. (2022). Ground-Based Thermal Imaging for Assessing Crop Water Status in Grapevines over a Growing Season. Agronomy.

[36] Galieni A., D’Ascenzo N., Stagnari F., Pagnani G., Xie Q., Pisante M. (2021). Past and Future of Plant Stress Detection: An Overview from Remote Sensing to Positron Emission Tomography. Front. Plant Sci..

[37] Bonanomi G., Battista Chirico G., Palladino M., Gaglione S.A., Crispo D.G., Lazzaro U., Sica B., Cesarano G., Tushar F.I., Sarker C. (2017). Combined application of photo-selective mulching films and beneficial microbes affects crop yield and irrigation water productivity in intensive farming systems. Agric. Water Manag..

[38] Wang L., Poque S., Valkonen J.P. (2019). Phenotyping viral infection in sweet potato using a high-throughput chlorophyll fluorescence and thermal imaging platform. Plant Methods.

[39] Rippa M., Pasqualini A., Curcio R., Mormile P., Pane C. (2023). Active vs. Passive Thermal Imaging for Helping the Early Detection of Soil-Borne Rot Diseases on Wild Rocket [*Diplotaxis tenuifolia* (L.) D.C.]. Plants.

[40] Rippa M., Del Regno C., Cappetta E., Russo A., Mele F., Curcio R., De Rosa A., Leone A., Mormile P., Ruocco M. (2023). Pulsed thermography as a reliable tool to detect presymptomatic stages of Botrytis cinerea infection in plants. ACS Agric. Sci. Technol..

[41] Pane C., Manganiello G., Nicastro N., Carotenuto F. (2022). Early detection of wild rocket tracheofusariosis using hyperspectral image-based machine learning. Remote Sens..

[42] McKinney H.H. (1923). Influence of soil temperature and moisture on infection of wheat seedlings by *Helminthosporium sativum*. J. Agric. Res..

[43] Elsayed S., Elhoweity M., Ibrahim H.H., Dewir Y.H., Migdadi H.M., Schmidhalter U. (2017). Thermal imaging and passive reflectance sensing to estimate the water status and grain yield of wheat under different irrigation regimes. Agric. Water Manag..

[44] Banerjee K., Krishnan P. (2020). Normalized Sunlit Shaded Index (NSSI) for characterizing the moisture stress in wheat crop using classified thermal and visible images. Ecol. Indic..

[45] Yuxuan W., Zia-Khan S., Owusu-Adu S., Miedaner T., Müller J. (2019). Early detection of *Zymoseptoria tritici* in winter wheat by infrared thermography. Agriculture.

[46] Romero-Bravo S., Méndez-Espinoza A.M., Garriga M., Estrada F., Escobar A., González-Martinez L., Poblete-Echeverría C., Sepulveda D., Matus I., Castillo D. (2019). Thermal Imaging Reliability for Estimating Grain Yield and Carbon Isotope Discrimination in Wheat Genotypes: Importance of the Environmental Conditions. Sensors.

[47] Gracia-Romero A., Kefauver S.C., Fernandez-Gallego J.A., Vergara-Díaz O., Nieto-Taladriz M.T., Araus J.L. (2019). UAV and Ground Image-Based Phenotyping: A Proof of Concept with Durum Wheat. Remote Sens..

[48] Ehrler W.L. (1973). Cotton leaf temperatures as related to soil water depletion and meteorological factors. Agron. J..

[49] Idso S.B., Jackson R.D., Reginato R.J. (1977). Remote-sensing of crop yields. Science.

[50] Ashfaq W., Fuentes S., Brodie G., Gupta D. (2022). The role of silicon in regulating physiological and biochemical mechanisms of contrasting bread wheat cultivars under terminal drought and heat stress environments. Front. Plant Sci..

[51] Ashfaq W., Brodie G., Fuentes S., Gupta D. (2022). Infrared Thermal Imaging and Morpho-Physiological Indices Used for Wheat Genotypes Screening under Drought and Heat Stress. Plants.

[52] Jackson R.D., Idso S.B., Reginato R.J., Pinter P.J. (1981). Canopy temperature as a crop water stress indicator. Water Resour. Res..

[53] Zhou Z., Majeed Y., Diverres Naranjo G., Gambacorta E.M.T. (2021). Assessment for crop water stress with infrared thermal imagery in precision agriculture: A review and future prospects for deep learning applications. Comput. Electron. Agric..

[54] Idso S.B., Reginato R.J., Jackson R.D., Pinter P.J. (1981). Foliage and air temperatures: Evidence for a dynamic “equivalence point”. Agric. Meteorol..

[55] Rodriguez D., Sadras V.O., Christensen L.K., Belford R. (2005). Spatial assessment of the physiological status of wheat crops as afected by water and nitrogen supply using infrared thermal imagery. Aust. J. Agric. Res..

[56] Jones H.G. (1999). Use of infrared thermometry for estimation of stomatal conductance as a possible aid to irrigation scheduling. Agric. For. Meteorol..

[57] Maes W.H., Achten W.M.J., Reubens B., Muys B. (2011). Monitoring stomatal conductance of Jatropha curcas seedlings under diferent levels of water shortage with infrared thermography. Agric. For. Meteorol..

[58] Mertens S., Verbraeken L., Sprenger H., De Meyer S., Demuynck K., Cannoot B., Merchie J., De Block J., Vogel J.T., Bruce W. (2023). Monitoring of drought stress and transpiration rate using proximal thermal and hyperspectral imaging in an indoor automated plant phenotyping platform. Plant Methods.

[59] Lindenthal M., Steiner U., Dehne H.W., Oerke E.C. (2005). Effect of downy mildew development on transpiration of cucumber leaves visualized by digital infrared thermography. Phytopathology.

[60] Elsayed S., Rischbeck P., Schmidhalter U. (2015). Comparing the performance of active and passive reflectance sensors to assess the normalized relative canopy temperature and grain yield of drought-stressed barley cultivars. Field Crop. Res..

